# Deletion of *Jdp2* enhances Slc7a11 expression in Atoh-1 positive cerebellum granule cell progenitors in vivo

**DOI:** 10.1186/s13287-021-02424-4

**Published:** 2021-06-29

**Authors:** Chia-Chen Ku, Kenly Wuputra, Kohsuke Kato, Jia-Bin Pan, Chia-Pei Li, Ming-Ho Tsai, Michiya Noguchi, Yukio Nakamura, Chung-Jung Liu, Te-Fu Chan, Ming-Feng Hou, Shigeharu Wakana, Yang-Chang Wu, Chang-Shen Lin, Deng-Chyang Wu, Kazunari K. Yokoyama

**Affiliations:** 1 Graduate Institute of Medicine, Regenerative Medicine and Cell Therapy Research Center, School of Medicine, Kaohsiung Medical University, Kaohsiung, 807 Taiwan; 2 Regenerative Medicine and Cell Therapy Research Center, Kaohsiung Medical University, 807, Koahsiung, Taiwan; 3 Department of Infection Biology, Graduate School of Comprehensive Human Sciences, The University of Tsukuba, Tsukuba, 305-8577 Japan; 4 Cell Engineering Division, Japan Mouse Clinic, RIKEN BioResource Research Center, Tsukuba, 305-0074 Japan; 5 Department of Gastroenterology, Cell Therapy and Research Center, Kaohsiung Medical University Hospital, Kaohsiung, 807 Taiwan; 6 Division of gastroenterology, Department of Internal Medicine, Kaohsiung University Hospital, 807, Kaohsiung, Taiwan; 7 Department of Obstetrics and Gynecology, Kaohsiung Medical University Hospital, Kaohsiung, 807 Taiwan; 8 Japan Mouse Clinic, RIKEN BioResource Research Center, Tsukuba, Ibaraki, 305-0074 Japan; 9 Department of Animal Experimentation, Foundation for Biomedical Research and Innovation at Kobe, Hygo, 650-0047 Japan; 10 Chinese Medicine Research and Development Center, China Medical University Hospital, Taichung, Taiwan

**Keywords:** Antioxidation, Cerebellum, Granule cells, Jun dimerization protein 2 (Jdp2), Reactive oxygen species (ROS)

## Abstract

**Background:**

The cerebellum is the sensitive region of the brain to developmental abnormalities related to the effects of oxidative stresses. Abnormal cerebellar lobe formation, found in Jun dimerization protein 2 (*Jdp2*)-knockout (KO) mice, is related to increased antioxidant formation and a reduction in apoptotic cell death in granule cell progenitors (GCPs). Here, we aim that Jdp2 plays a critical role of cerebellar development which is affected by the ROS regulation and redox control.

**Objective:**

*Jdp2*-promoter-Cre transgenic mouse displayed a positive signal in the cerebellum, especially within granule cells. *Jdp2*-KO mice exhibited impaired development of the cerebellum compared with wild-type (WT) mice. The antioxidation controlled gene, such as cystine-glutamate transporter Slc7a11, might be critical to regulate the redox homeostasis and the development of the cerebellum.

**Methods:**

We generated the *Jdp2*-promoter-Cre mice and *Jdp2*-KO mice to examine the levels of Slc7a11, ROS levels and the expressions of antioxidation related genes were examined in the mouse cerebellum using the immunohistochemistry.

**Results:**

The cerebellum of *Jdp2*-KO mice displayed expression of the cystine-glutamate transporter Slc7a11, within the internal granule layer at postnatal day 6; in contrast, the WT cerebellum mainly displayed Sla7a11 expression in the external granule layer. Moreover, development of the cerebellar lobes in *Jdp2*-KO mice was altered compared with WT mice. Expression of Slc7a11, Nrf2, and p21^Cip1^ was higher in the cerebellum of *Jdp2*-KO mice than in WT mice.

**Conclusion:**

Jdp2 is a critical regulator of Slc7a11 transporter during the antioxidation response, which might control the growth, apoptosis, and differentiation of GCPs in the cerebellar lobes. These observations are consistent with our previous study in vitro.

**Supplementary Information:**

The online version contains supplementary material available at 10.1186/s13287-021-02424-4.

## Background

The cerebellum is the most vulnerable region of the brain to developmental abnormalities related to the effects of oxidants. Reactive oxygen species (ROS) act in various ways within the cerebellum, including effects on cell proliferation and cell death based on cell fate determination [1]. The intracellular levels of ROS depend on the balance between ROS production and antioxidation. Glutathione (GSH) is one of the major antioxidants within cells [2]. The ratio of reduced to oxidized glutathione disulfide (GSSG) (GSH/GSSG) is a critical intracellular redox index state [3]. GSH levels are controlled by large number of genes, including the cystine–glutamine antiporter (Xc-) systems, which include solute carrier family 7, member 11 (Slc7a11) [4–6], solute carrier family 3, member 2 (Slc3a2) as a binding partner, and CD44v [7]. The Xc-system mediates cystine transport, which is rapidly reduced to cysteine and used for biosynthesis of protein and GSH, as part of the major redox balance [4].

During development, cerebellar cell subtypes are produced from two distinct primary germinal centers, the ventricular zone (VZ) and the rhombic lip (RL), in a sequential manner [8]. The VZ is characterized by the expression of Mash1, neurogenins, and Ptf1α [9], while the RL is defined by the expression of Atoh1 and Pax6 [10]. During postnatal development, the VZ delaminates to give rise to the secondary germinal center [11]. In contrast, the RL progenitors move above the subapical layer tangentially to produce the external granular layer [12]. The VZ progenitors produce all the GABAergic neurons and glial cells of the cerebellum, whereas the RL progenitors generate all the glutamatergic neuronal subtypes [13].

The Jun dimerization protein 2 (Jdp2), which is a member of the activator protein (AP)-1/activation transcription factor (ATF) family of transcription factors, participates in the repression of transcription via multiple mechanisms [14–16]. In addition, in mouse embryonic fibroblasts (MEFs), Jdp2 is a component of the Nrf2–MafK complex in the control of antioxidative response elements (ARE) and ROS [17]. Jdp2 interacts with Nrf2 and MafK to form a complex and binds to the core sequence of AREs, and then activates the transcription of target genes such as antioxidant- and detoxification-related enzymes [17]. As a result, Jdp2 inhibits ROS generation and generates more reduced conditions in MEFs. However, the developmental role of Jdp2 in cerebellar neurons remains to be solved. Our previous study demonstrated that Jdp2 plays a role to control the ROS production and GSH production through the higher expression of Scl7a11 in the granule cell progenitors (GCPs) [18]. Thus, we seek for this relationship of Jdp2 and Scl7a11 in the mouse model.

Therefore, in the current study, we used in vivo immunohistochemistry to demonstrate the role of Jdp2 in antioxidation via effects on Slc7a11. The data suggest that these actions contribute to the control of cerebellar GSH content and cerebellar lobe formation.

## Materials and methods

### Animals, organs, and cells

Animal welfare guidelines published by the Animal Care Committee of the RIKEN BioResource Research Center (BRC) in Japan (Kiteisv.intra.riken.jp/JoureiV5HTMLContents/act/print/print110000514.htm), the National Laboratory Animal Center (NLAC) (106022), and the Kaohsiung Medical University in Taiwan (106189; 107128; 108244), were adhered to for the care of laboratory animals [17]. The cerebellum and the cerebellar granule cell progenitors (GCPs) were derived from six (P6) and 24 (P24) days postnatally and from adult mice 8, 9, 12, 15, 16 and 22 weeks of age. All animal experiments were conducted according to these approved guidelines. Various organs were isolated from the *Jdp2*-KO mice (RIKEN Modified SHIRPA; https://ja.brc.riken.jp/lab/jmc/shirpa/en/) and evaluated for LacZ expression. The strategy used to produce the *Jdp2-*KO mouse has been described elsewhere [19–21]. Primary granule cell progenitors (GCPs) were prepared as described [18].

### Preparation of Jdp2-promoter-Cre mice

The 2.5 kb-DNA insert contained a 5′-end untranslated region of the *Jdp2* gene, the transcription initiation ATG codon of *Jdp2* mRNA, a nuclear localization signal fragment of the *Cre* gene, an internal ribosome entry site DNA fragment, and the NLS-LacZ (β-galactosidase; β-Gal) DNA, followed by the polyadenylation motif (poly A) DNA fragment of SV40 [19]. The *Swa*1 linearized DNA was microinjected into fertilized FVB/N eggs. Twenty-five transgenic mouse lines (F0) were obtained and further crossed with ROSA26R reporter [22] or ZEG (TCTB-βgeo-green fluorescent protein [GFP]) reporter [23] mice. F1 offspring were characterized by staining of X-gal or GFP, and the genotype of these F1 founders was tested by PCR. PCR genotyping was carried out with the following three primers: Primer 1, 5′-GGGTTAAGTGGAATCAGTTCTGCTC-3′; Primer 2, 5′-GGTTCAGGGGGAGGTGTGGGAGG-3′ (SV40 polyA); and Primer 3, 5′-GGAAGGCGATCCCATAGGAAGAG-3′. The WT (Primers 1 and 3) and mutant DNA fragments (Primers 1 and 3) were 688 and 420 bp, respectively (GenBank, AB034697, BC019780 and AB07743). C57BL6/J × 129v, 129v, and C57BL6/J congenic backgrounds were available from the RIKEN BRC, Tsukuba, Japan, and the NLAC in Taipei, Taiwan.

### Tissue preparation and hematoxylin and eosin (H&E) staining

Mice were anesthetized with ketamine (10 mg/ml) and were then perfused with 10 mM phosphate-buffered saline (PBS; pH 7.4), followed by 4% (w/v) formaldehyde in PBS. Whole brains were removed, fixed by immersion in 4% (w/v) formaldehyde in 10 mM PBS overnight at 4 °C, and subsequently cryoprotected in 30% (w/v) sucrose in 10 mM PBS. Tissue samples were embedded in paraffin wax and sections cut (4–5 μm) for staining with H&E [24]. Tissue H&E staining was examined using an Olympus CKX41 microscope (Olympus, Tokyo, Japan), and images were scanned by a TissueFax microscope (TissueGnostics, Vienna, Austria). The area of cerebellar lobules was quantified, the nuclei (stained with hematoxylin) were counted, and the cytoplasm (stained with eosin) in lobules I–V, VI–VII, VIII–IX, and X–XI was quantified using HistoQuest tissue analysis software (TissueGnostics). The angularity of the cerebellum was calculated from the angles from the top of the cerebellum to each lobe and was indicated by angles (A)_1–4_, according to the equation [(A_3_/A_4_) / (A_2_/A_1_)] × 100%.

### Immunohistochemistry

Tissue sections were rehydrated in 10 mM PBS, and antigens were retrieved in sodium citrate buffer (10 mM sodium citrate, 0.05% Tween 20, pH 6.0) by heating at 121°C, for 15 min. Endogenous peroxidase activity within the sections was terminated by treatment with 3% H_2_O_2_ in 10 mM PBS for 15 min, followed by rinsing with PBS-T (PBS plus 1% Triton X-100). Sections were incubated at room temperature for 20 min with blocking solution (10% normal serum in PBS-T) and subsequently incubated at 4°C overnight with primary antibodies in a humid chamber. After washing with PBS-T, sections were treated with secondary antibodies and processed for 3,3′-diaminobenzidine immunohistochemistry using ChemMate™ DAKO EnVision™ Detection kits (K5007; Dako, Glostrup, Denmark). Slides were dehydrated, and coverslips were applied. Primary antibodies were visualized with Atoh1 (1:200; Chemicon; AB5692), Slc7a11 (1:200; Novus Biologicals; nb300-318), p21^Cip1^ (1:50; Santa Cruz Biotechnology; sc-397), Nrf2 (1:50; Bioworld Technology; BS1258), cytochrome C (1:200; Cell Signaling Technology; CST#4272), and BrdU (1:200; Cell Signaling Technology; CST#5292). Slide images were scanned and saved using a TissueFax microscope (TissueGnostics, Vienna, Austria). The incorporation of BrdU (SI-B5001, Sigma-Aldrich) into newborn mouse pups was conducted according to the method of Kadam et al. [25]. We prepared 10 mg/ml BrdU in DMSO solution and 20 μl (30 mg/kg at a concentration 30 mg/mL) was injected subcutaneously into the dorsal neck fold of postnatal day 6 (P6) pups. After 60 min, we analyzed the cerebellum by staining for BrdU according to the BrdU labeling and detection protocol (Thermo Fisher Scientific, Invitrogen). The images were quantified by using the open access software Fiji/ImageJ analysis software (https://imagej.net/Fiji) [26].

### Western blotting

Western blotting was performed as described [18, 27–29]. Briefly, cerebella were homogenized in ice-cold Neuronal Protein Extraction Reagent (N-PER; Thermo Fisher Scientific), containing Halt Phosphatase Inhibitor Cocktail (Thermo Fisher Scientific), following the manufacturer’s protocol. Protein lysates (20–50 μg) were resolved on NuPAGE 4–12% Bis-Tris protein gels and transferred to Immobilon-P transfer polyvinylidene difluoride (PVDF) membranes (0.45 μm; Merck) for 1 h at 100 V (fixed) at 10°C, using a TE22 Hoefer transfer system (Hoefer Inc., Holliston. MA, USA). To monitor the levels of transferred proteins, blots were stained with Ponceau S (Merk). PVDF membranes were then incubated with the primary and secondary antibodies (see Supplementary Table 1) and analyzed using a ChemiDoc XRS_Plus instrument (Bio-Rad, Hercules, CA, USA).

### RNA sequencing, gene clustering, and gene categorization

RNA sequencing was conducted using a Genome Analyzer IIX System (Illumina, San Diego, CA, USA) and employing the 50-bp single-end protocol by Welgene Biotech (Taipei, Taiwan) as described [28, 29]. Sequencing libraries were constructed by TruSeq RNA Sample Prep kits v2 and sequenced on an Illumina GAIIx platform using the 50-bp single-end protocol. The sequence was directly determined using sequencing-by-synthesis technology with the TruSeq SBS Kit. Raw sequences were generated by the Illumina GA Pipeline software, CASAVA. The sequences obtained went put through a filtering process using ConDeTri [30] to obtain qualified reads, which were investigated and estimated by TopHat/Cuffdiff [31]. The Human Genome Build 19 and gene features were retrieved from the Ensemble database and used for processing. The gene expression levels were calculated as reads/kilobase of transcript/million mapped reads (RPKM). Differentially expressed genes were filtered using an RPKM ≥ 0.3, a fold change ≥ 2, and with a *p* value < 0.05. RNA sequencing data were deposited in the NCBI Bioproject Database (http://www.ncbi.nlm.nih.gov/bioproject) with the accession numbers SUB3541857, SUB3541902, SUB3541913, and SUB3541945.

Hierarchical clustering of the genes was performed as follows: first, gene-level normalization was performed by transforming the RPKM of each gene of each sample to a Log2 median-centered ratio; subsequently, clustering was obtained by Euclidean distance and complete linkage settings; finally, a heatmap was generated by coloring each gene on the Log2 median-centered ratio. To convert gene symbols to Ensemble gene accessions, the unique gene symbols of each topic were mapped to Ensemble official symbols.

### The Cancer Genome Atlas/cBioPortal analysis

Large-scale cancer genomics projects like The Cancer Genome Atlas [32] are generating an overwhelming amount of cancer genome data from different technical platforms. The cBioPortal for Cancer Genomics (http://cbioportal.org) provides a Web resource for exploring, visualizing, and analyzing multidimensional cancer genomics data. The portal reduces molecular profiling data from cancer tissues and cell lines into readily understandable genetic, epigenetic, gene expression, and proteomic events [33, 34]. This accelerates the translation of genomics data into the identification of cascades, therapies, and clinical trials. The cBioPortal (http://www.cbioportal.org/faq#how-do-i-cite-the-cbioportal) data were accessed, and we surveyed Sla7a11 gene mutation maps in brain tumors. Five thousand nine hundred fifty-two patients and 6166 samples from 20 studies were grouped for each item, such as apoptosis and cell cycle progression pathways [32, 33]. We used the cBioPortal database to search for brain-related papers, and we used the filtered 20 papers to query the genes TP53, CDKN1A, CDKN2A, CCNA2, CCND1, SCL7A11, and NFE2L2 in a total of 6166 samples.

### Statistical analysis

Data are presented as mean ± standard error of the mean (SEM). Statistical comparisons between experimental conditions were carried out using GraphPad Prism 5.0 (GraphPad Software, San Diego, CA, USA). For multiple comparisons, a one-way ANOVA followed by a Tukey post hoc test or a two-way ANOVA with a Bonferroni post hoc test was used. An unpaired, two-tailed Student’s *t* test was used to compare the control and treatment groups. A Mann–Whitney nonparametric median statistical test was used for analyses of cell areas. All differences were designated as statistically significant at *P* < 0.05.

## Results

### Monitoring of the *Jdp2* promoter in the mouse brain

**Fig. 1:**
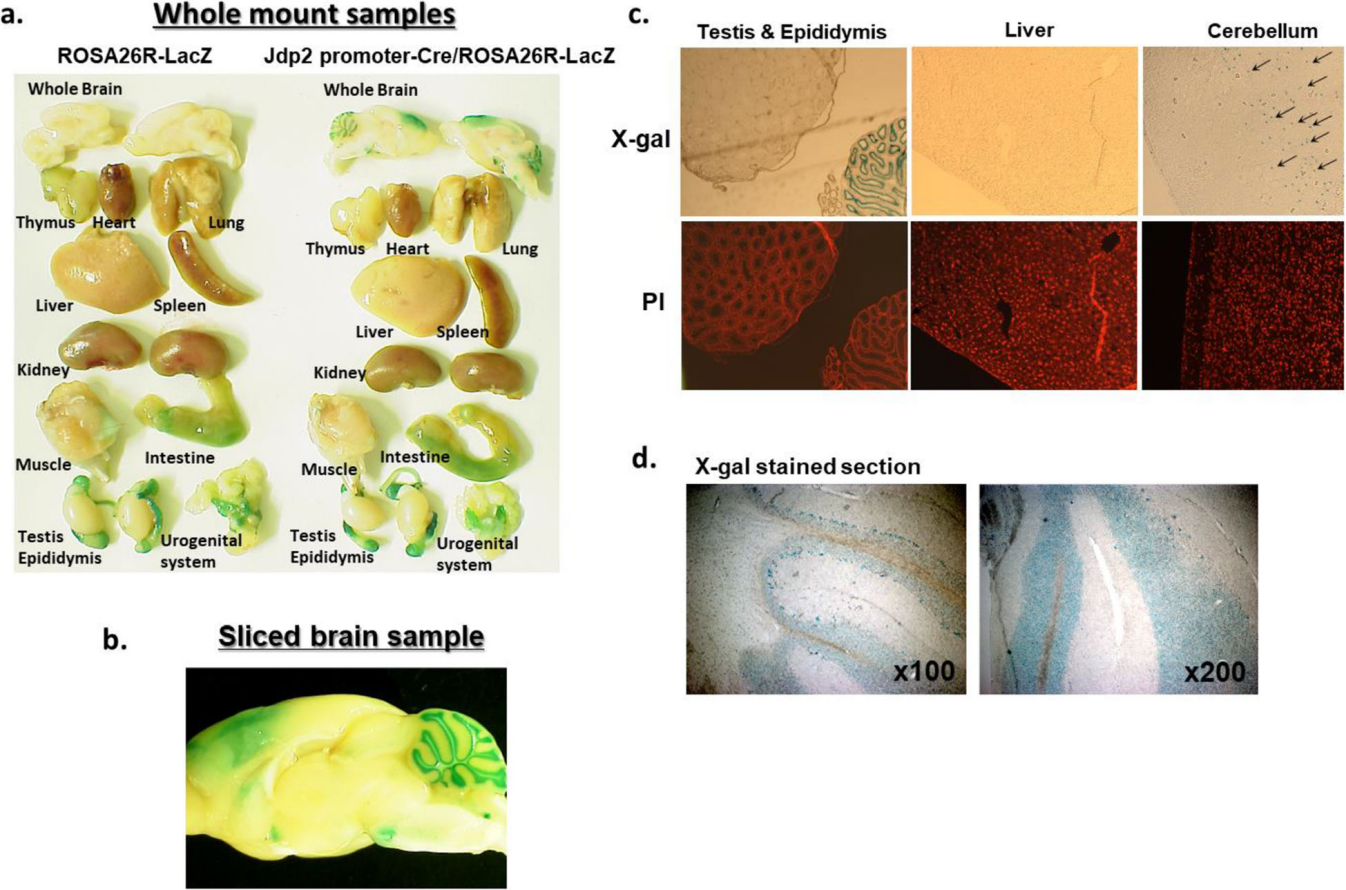
Development of cerebellar lobes and granule cells in *Jdp2*-promoter-Cre mice. **a** β-Gal staining of the *Jdp2* promoter-Cre/ROS26R-LacZ adult mouse revealed signals in the various organs. WT 129-C57/BL6J ROSA26R-LacZ and *Jdp2*-promoter-Cre/ROSA26R-LacZ male mice were compared (8–22 weeks), and the brain displayed strong β-Gal staining in WT 129-C57/BL6J *Jdp2*-promoter-Cre/ROSA26R-LacZ male mice at P24. **b** β-Gal staining was mainly detected in the cerebellum and some parts of the cerebrum of WT 129-C57/BL6J *Jdp2*-promoter-Cre/ROSA26R-LacZ male mice at P24. **c** The β-Gal staining was detected in testis, epididymis, and cerebellum, but not in the liver in WT 129-C57/BL6J *Jdp2*-promoter-Cre/ROSA26R-LacZ male mice at P24. Arrows indicate β-Gal-stained cells. Propidium iodide (PI) was used as a nuclear DNA counterstain. **d** β-Gal-stained cells in the cerebellum from WT 129-C57/BL6J *Jdp2*-promoter-Cre/ROSA26R-LacZ male mice at P24 were viewed under bright field at × 100 and × 200 magnification. The data were shown as one example among 9 male mice at P24

Previously, we generated a double-transgenic *Jdp2-*promote*r*-Cre/ROSA26R-LacZ and a *Jdp2-*promote*r*-Cre/Z/EG mouse strain (SHIRPA S455; http://ja.brc.riken.jp/lab/jmc/shirpa/en/) and reported some initial findings [18–21]. The *Jdp2*-promoter-Cre/ROSA26R-LacZ mice expressed LacZ signal in the brain, especially in the cerebellum (Fig. 1a, b). The LacZ signal was also detected in cerebrum, testis, and epididymis, but not in the liver (Fig. 1c). In the brain, the LacZ signal was found in cerebellar granule cells (Fig. 1d). The Cre activity detected in the cerebellar granule cells was derived from the *Jdp2* promoter (Fig. S2b in [19]). These data indicate that the *Jdp2* promoter is active in the cerebellum, especially in the area rich in granule cells. Previously, we also characterized primary granule cells that expressed LacZ signal and GFP signal in vitro [21]. Furthermore, we compared the morphology and size of cerebellar lobes in WT and *Jdp2*^–/–^ 129-C57BL/6 J mice.

### Abnormal development in the cerebellum lobe of Jdp2-KO mice

**Fig. 2:**
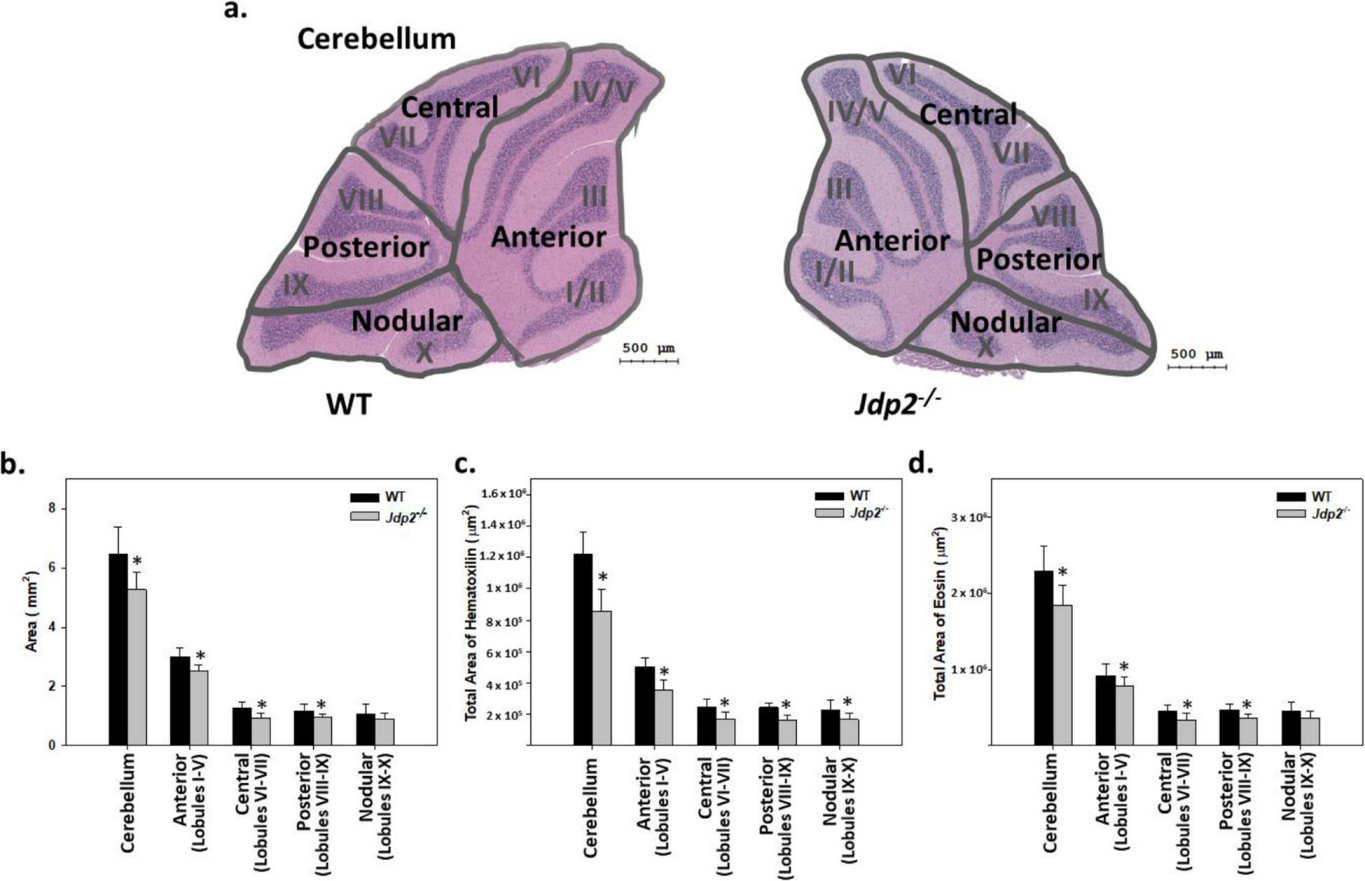
Hematoxylin and eosin staining of cerebella from WT 129-C57/BL6J and *Jdp2*-KO 129-C57/BL6J adult mice. **a** The volume of the cerebellum was reduced to 70–80% of that in WT mice, and lobes I–V and VI–VIII of the cerebella in *Jdp2*-KO mice were smaller than those in WT cerebella. **b**–**d** Lobe-size, hematoxylin-stained area, and eosin-stained area were quantified as described in the “Materials and methods” section. The experiments were repeated 5 times using 9 mice at 8-22 weeks. The section number was more than 16, the sample number was 3 mice for WT 129-C57/BL6J and 6 mice for *Jdp2*-KO 129-C57/BL6J mice, the lobes IV, and V/VI were analyzed. * *p* < 0.05

The cerebellum is morphologically divided into a central vermis flanked by lateral hemispheres. Both the vermis and the hemispheres are subdivided into a series of parallel fissures that define a conserved pattern of folia [35]. In the current study, we compared the morphology of the cerebellum in WT and *Jdp2*-KO mice. Previous reports suggested that there was no significant loss or excess of cerebellar lobes in *Jdp2*-KO mice relative to WT mice [18, 19]. However, in a careful reexamination, we identified morphological differences; cerebella in *Jdp2*^–/–^ 129-C57BL/6 J mice were significantly smaller than those in WT 129-C57BL/6 J mice, according to hematoxylin and eosin staining (H&E staining) [36, 37]. According to the RIKEN Modified SHIRPA (https://ja.brc.riken.jp/lab/jmc/shirpa/en/), body weight, tail length, and the ratio of tail length to body length were smaller in *Jdp2*-KO mice relative to WT mice (unpublished data). In this study, we H&E stained the cerebella of adult WT and *Jdp2-*KO mice and measured the size of lobes using the HistoQuest 7.0 program (TissueGnostics). We found that the anterior part and the remaining three parts were smaller in *Jdp2*-KO than in WT cerebellar lobes. Thereafter, we divided the vermis of the cerebellum into four regions, anterior (lobes I–V), central (lobes VI–VII), posterior (lobes VIII–IX), and nodular (lobes X-XI) (Fig. 2a) to examine the cerebellar morphology and area of each region of WT and *Jdp2*-KO mice. In *Jdp2*-KO mice, lobules I–V were 20–35% smaller, lobules VI–VII were 20–28% smaller, and lobules VIII–IX were 17–20% smaller than those of WT mice (Fig. 2b–d). Male and female mice did not differ in their cerebellar size (data not shown).

**Fig. 3:**
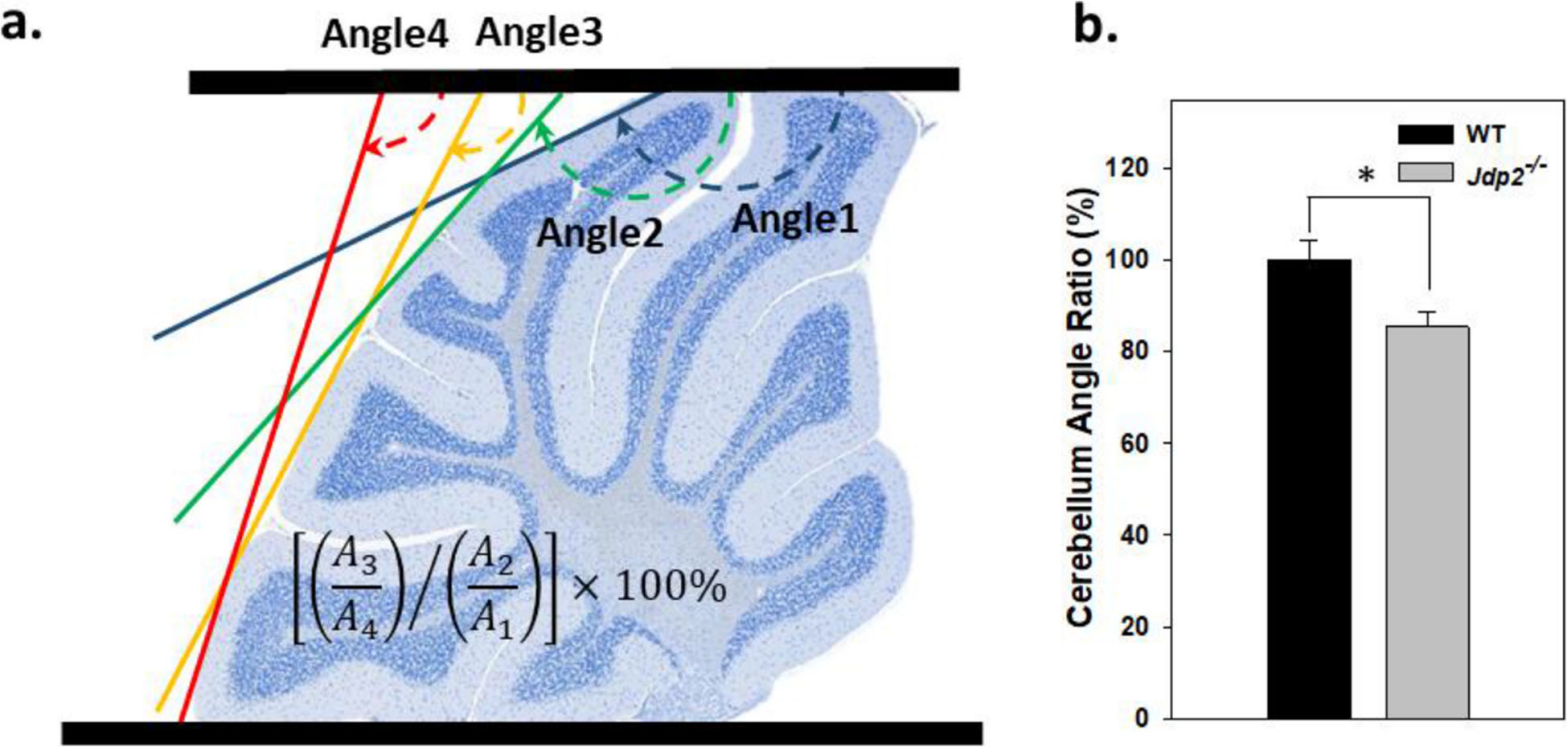
Calculation of the cerebellum angles in each lobe. **a** Schematic presentation of each angle of cerebellum, I–XI from WT129-C57/BL6J mice at 12 weeks. **b** The angle ratio of cerebellar lobes from regions VI–VIII of *Jdp2*-KO mice was ~ 20% less than that in the same lobes of WT mice; the experiments were repeated 5 times using mice at 12–22 weeks. The section number was 14; the sample number was 3 mice for WT 129-C57/BL6J and 9 mice for *Jdp2*-KO 129-C57/BL6J male mice; the lobes IV and V/VI were analyzed. * *p* < 0.05

The mouse cerebellar angle ratio was calculated (Fig. 3a, b). The upper perpendicular line of lobes V and VI and the lower perpendicular line of lobes IX and X were fixed. This upper perpendicular line and the angular line along lobe VI were drawn and the angle between these two lines was measured as A1. The angle between the upper perpendicular line and the angular line of lobes VI–VII was measured and set as A2. Similarly, the angle between the upper perpendicular line and the angular line of lobes VIII–IX, and the angular line of lobe IX were measured and set as A3 and A4, respectively. The final angle ratio was then calculated using the equation [(A_3_/A_4_) / (A_2_/A_1_)] × 100%. According to this estimation, the cerebellar angle ratio was lower in *Jdp2*-KO mice that in WT mice (Fig. 3a, b).

**Fig. 4:**
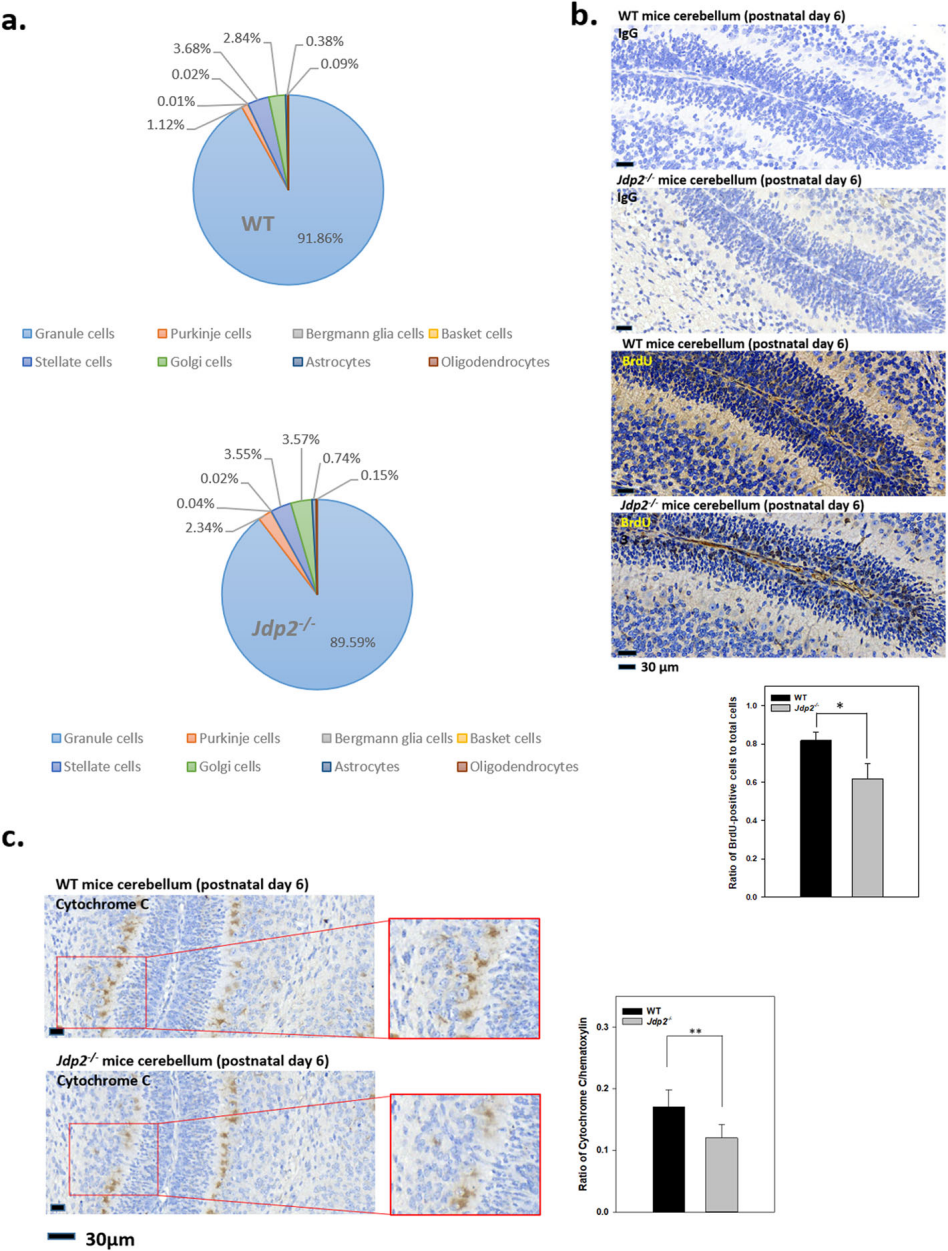
Comparative expression of cell type-specific marker genes in primary granule cell progenitors (GCPs) derived from P6 mice cerebellum and the immunostaining of BrdU-incorporated cells and apoptotic cells between WT and *Jdp2-*KO mice at P6. **a** Comparative expression of marker genes in the cerebellum between WT and *Jdp2-*KO mice. The granule cells were estimated by the expressions of *Atoh-1*, *Zic1*, *Pax6*, *NeuroD1*, and *Lhx9*; and Purkinje cells were marked by the expressions of *Calbindin 1*, *2*, *Doc2b* and *Pcp4*. Bergman glia were estimated by expressions of *Sept4* and *Cdf10*, basket cells are represented by *Cck*, and stellate cells by *Gria2*, astrocytes by *Cd44*, and oligodendrocytes by *Oligo2*. These expression patterns were calculated as total RNA expression and then summarized as a percentage of the relative RNA expression profiles in the cerebellum. The expression profiles of each cell type seem not to be big difference, but the expression of granule cells was higher in WT then *Jdp2*-KO mice. In contrast, the Purkinje cell expression profile in *Jdp2*-KO mice was higher than that in WT mice. **b** Comparative BrdU incorporation was evaluated by immunohistology of the cerebellum in WT and *Jdp2*-KO mice at P6. Expression of BrdU in cerebellum was 1.3-fold higher in WT than *Jdp2*-KO mice (80% vs 60%). Immunohistochemistry was performed as describe in the “Materials and methods” section. The section number was 10; the sample number was 5 mice for WT 129-C57/BL6J and 5 mice for *Jdp2*-KO 129-C57/BL6J mice; the lobes IV and V/VI were analyzed. *p < 0.05. **c** Expression of cytochrome C was examined by immunohistology of the cerebellum in WT and *Jdp2*-KO male mice at P6. Cytochrome C in cerebellum was higher in WT than *Jdp2*-KO mice. The section number was 14, and the sample number was 7 mice for WT 129-C57/BL6J and 7 mice for *Jdp2*-KO 129-C57/BL6J mice; the lobes IV and V/VI were analyzed. Immunohistochemistry was performed as describe in the “Materials and methods” section. **p < 0.01

In studies of the early development of the cerebellum, it is reported that GCPs represented > 80% of the cell population within the external germinal layer [35]. We confirmed this report in the present study. Carter et al. [38] conducted a single-cell, RNA sequencing study, which revealed that the granule cell population in the cerebellum represented > 80% of the total cell population. In line with this report, we have studied the expression levels of cell-type marker genes of the cerebellum. Granule cells were estimated by the expression of *Atoh1*, *Zic1*, *Pax6*, *NeuroD1*, and *Lhx9*; Purkinje cells were detected by the expression of *calbindin 1*, *2*, *Doc2b*, and *Pcp4*. Similarly, Bergmann glia were detected by the expression of *Sept4* and *Cdf10l*; basket cells by the expression of *Cck*; stellate cells by the expression of *Gria2*; astrocytes by the expression of *Cd44*; and oligodendrocytes by the expression of *Oligo2*. These values (*p* < 0.05) were summarized relative to the total number of cells (Fig. 4a) and resembled the data from a recent single-cell RNA sequencing study [38]. When expression levels of each representative cell population of *Jdp2*-KO GCPs were compared with those in WT GCPs, the expression of granule cell marker genes was less than that in WT GCPs; in contrast, the expression levels of marker genes for Purkinje cells, astrocytes, and oligodendrocytes were 1.7–2.0-fold higher in *Jdp2*-KO GCPs than those in WT cells.

RNA sequencing results from WT and *Jdp2*-KO GCPs derived from P6 mice indicated that > 95% of the cells isolated from cerebella were positive for Atoh1, which is known to be indispensable for maintaining GCPs in the immature state in the external granule layers. Loss of Atoh1 results in precocious differentiation from GCPs to GCs [39]. Thus, the precise regulation of Atoh1 protein levels by transcriptional activation and subsequent degradation is required for the proper development of GCP2 to GCs [40]. However, both the number of Atoh1-positive cells and the expression level of Atoh1 were reduced in *Jdp2*-KO GCPs compared with those in WT GCPs [18]. Thus, these observations suggest that Jdp2 is required for the normal proliferation and differentiation of GCPs and subsequently for cerebellar lobe development. The cell numbers stained with anti-Atoh1 as the GCP marker and anti-Calbindin as Purkinje cells marker as well as Slc7a11, p21^Cip1^ and Nrf2 as antioxidation markers were compared in WT and *Jdp2*-KO cerebellums. The numbers of Slc7a11-positive cells and p21^Cip1^- positive cells were higher in *Jdp2*-KO cerebellum than WT cerebellum. In this case, the numbers of Atoh1-positive cells in *Jdp2*-KO cerebellum were lower than that of WT cerebellum, but the numbers of Calbindin-positive cells were higher in *Jdp2*-KO than that of WT cerebellum. These results indicate that Jdp2 controls the expansion of Atoh1-stained GCPs as well as that of Calbindin-stained Purkinje cells (Fig. S1; refs 18, 19). This observation is examined further.

The cancer cohorts originated from the cerebellar granule cells and the precise mapping of the medulloblastomas was reported previously [41, 42]. Four distinct nonoverlapping variants were identified: WNT, Sonic hedgehog (SHH), and group C and group D. The RNA sequences of *Jdp2* WT and *Jdp2*-KO GCPs have been compared (Fig. S2 and [18]). The expression profile of cultured *Jdp2* GCPs at day 1 and day 7, resembled to the expression profile of the group of the SHH pathway.

Studies of the incorporation of BrdU demonstrated that incorporation in the lobe regions of WT cerebellum was ~ 1.3-fold higher than in *Jdp2*-KO cerebellum from P6 mice (Fig. 4b). To further investigate the role of Jdp2 in GCP control, the apoptosis of GCPs was examined by immunostaining of anti-cytochrome C (Fig. 4c) and a TUNEL assay [18] previously. We found that the number of cytochrome C-positive cells was ~ 1.4-fold higher in WT cerebellum than it was in *Jdp2*-KO cerebellum in P6 mice (Fig. 4c). In addition, we already reported that the respective percentages of apoptotic cells in WT and *Jdp2*-KO GCPs were 22% and 11% [18]. Furthermore, results from annexin V assays indicated a decrease in apoptotic cell death in *Jdp2*-KO GCPs [18].

### Increased antioxidation activity and Slc7a11 expression in Jdp2-KO cerebellum

In studies to determine whether the cellular response to oxidative stress was altered by *Jdp2* deficiency, we reported that the ROS activity was 50% lower in *Jdp2*-KO GCPs than in WT GCPs [18]. In contrast, both the GSH content and the GSH/GSSG ratio were higher in *Jdp2*-KO GCPs than in WT GCPs; and these changes might be responsible for the reduction in oxidative stress observed in *Jdp2*-KO GCPs [18].

**Fig. 5:**
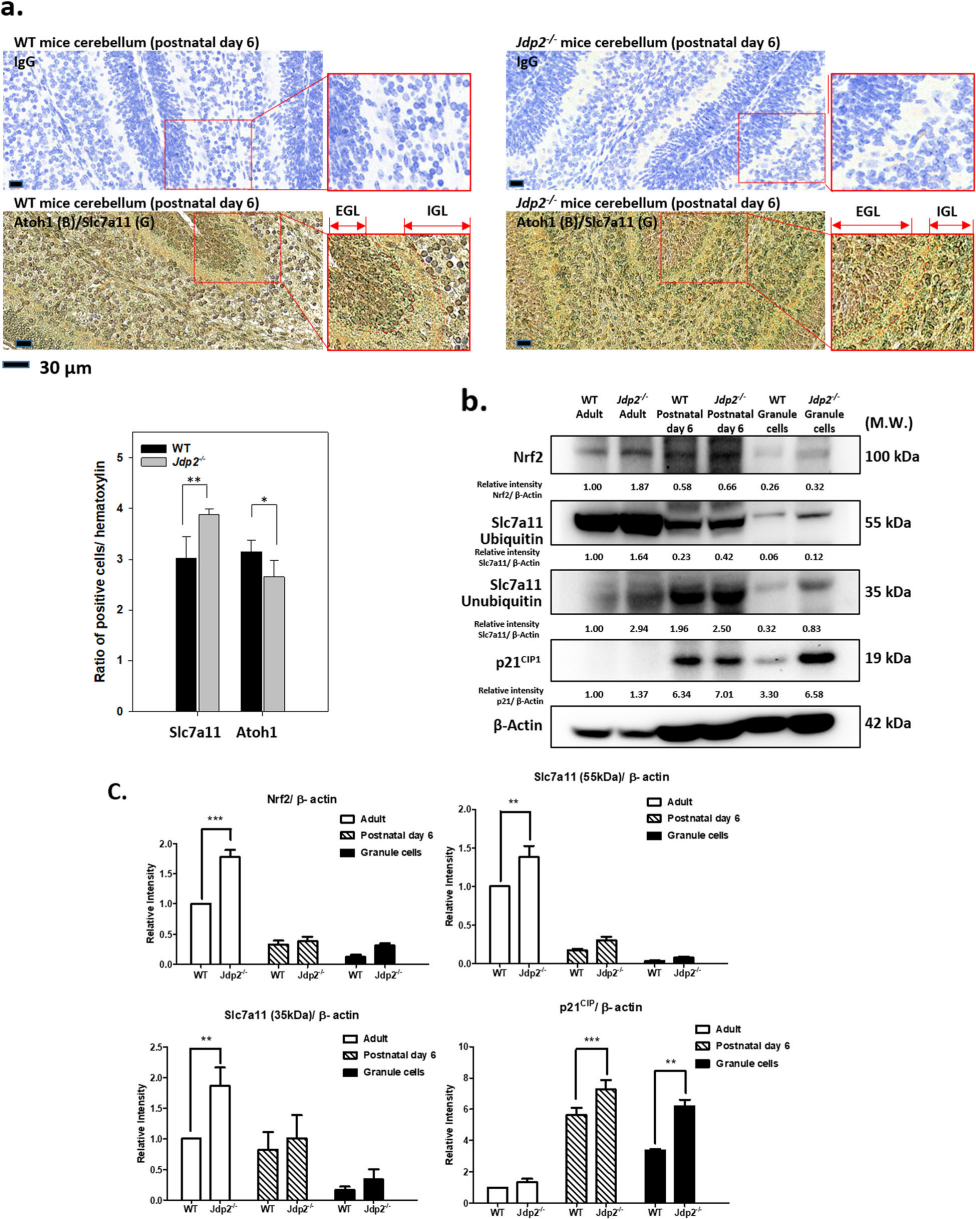
Comparative immunostaining of the cerebellum between WT and *Jdp2*-KO mice. Immunohistochemical analysis of cerebellum in WT and *Jdp2*-KO mice at P6 was performed using anti-Atoh1, and Slc7a11 proteins. **a** Cerebellum from *Jdp2*-KO and WT mice were stained for Atoh1. The ratio of Atoh1-stained cells to the total number of cells was estimated using the immunohistochemistry-stained-slides as described previously [18]. Extended Figure-panels in Atoh1 (brown color) and Slc7a11 (green color) IHC sample were shown as the marks inside and outside to focus upon the staining in GCPs and Purkinje cells [43]. The section number was 6; the sample number was 3 mice for WT 129-C57/BL6J and 3 mice for *Jdp2*-KO 129-C57/BL6J mice. * *p* < 0.05, ** *p* < 0.01. **b** Comparative expression of antioxidation-related proteins in WT and *Jdp2*-KO cerebellum from mice at 9 weeks, P6, and GCPs (n = 5). The western blotting data from GCPs derived from WT and *Jdp2*-KO mice were the same to those previously reported [18]. Slc7a11 35 kDa protein was an unubiquitinated form and Slac7a11 55 kDa protein is ubiquitinated form as previously reported [18]. The right lane indicates the apparent molecular weight. The relative expression based on β-actin was calculated in the lower panel of each. Western blotting was carried out as described in the “Materials and methods” section. **c**. Statistical analysis of the data from Western blotting as sown in panel b. n = 5. The statistics analysis was performed as describe in the “Materials and methods” section. ** *p* < 0.01, *** *p* < 0.005

To elucidate the mechanism responsible for the increased production of GSH in *Jdp2*-KO GCPs, we performed immunohistochemistry staining and western blotting analysis to measure the levels of Slc7a11 protein, detected in a 55 kDa ubiquitinated form and a 35 kDa unubiquitinated form [18], and found increased levels *Jdp2*-KO GCPs, indicating that the intake of cystine might be increased for GSH biosynthesis [18]. We stained the cerebellum for Sla7a11 (Fig. 5a) to compare WT and *Jdp2*-KO cerebellum at P6. Slc7a11 staining displayed a 1.27-fold stronger signal in *Jdp2*-KO cerebellum than in WT cerebellum, although the Atoh1-stained cells in *Jdp2*-KO cerebellum were 0.84-fold lower in WT cerebellum (Fig. 5a). Moreover, Atoh1 and Sla7a11 appeared colocalized mainly in the external granule layers in WT cerebellum. In contrast, these signals were clearly detected in the internal granule layers in *Jdp2-*KO cerebellum (Fig. 5a and Fig. S1a).

Previously, we reported that the rate of cystine uptake and intracellular cystine concentrations were increased in *Jdp2*-KO GCPs as compared with WT GCPs [18]. These data indicate that *Jdp2*-KO GCPs in cerebellum have an increased antioxidant activity. To confirm this observation, we performed staining of the cerebellum using antibodies against Nrf2, and p21^Cip1^ because we previously found that Jdp2-Nrf2-p21^Cip1^ were complexed in the nuclei of the PGCs [18]. Here, the staining intensity of p21^Cip1^ and Nrf2 was stronger in the *Jdp2*-KO cerebellum than it was in WT cerebellum from P6 mice based on the imaging software (Fig. S1a). However, western blotting showed that the p21^Cip1^ protein was not found in adult cerebellum (Fig. 5b). Immunostaining of p21^Cip1^ in the *Jdp2*-KO mouse cerebellum at P6 was 1.25-fold higher than it was in WT mice. However, our western blotting data revealed no such significant difference in the cerebella from P6 WT and *Jdp2*-KO mice (Fig. 5c). At this stage, we cannot rule out the possibility that other cells beside PGCs might have higher levels of p21^Cip1^ in cerebellum. Even so, our data suggest that the antioxidation state is higher in the cerebellum of *Jdp2*-KO mice than it is in WT mice.

The Cancer Genome Atlas (TCGA) cohort was collected from human cancer patients with a mutation in the *Slc7a11* gene (see the cBioPortal for Cancer Genomics in [31–33]; Fig. S3). We searched 6,166 samples from 5952 patients and found that Slc7a11 was involved in a series of genes associated with cell cycle progression and apoptosis (Fig. S4 and [18]), such as *TP53*, *CDKN2A*, *CDKN1A*, *CCND1*, and *NFE2L2* (Table S1). The mutation frequency of the cancer genes *TP53*, *CDKN2A*, *CDKN1A*, *CCND1*, and *NFEL2* was significantly higher than that of normal genes *Slc7a11* and *CCNA2* (*p21*^*Cip1*^) and their co-occurrence in cancer patients is well established (Table S2 and ref. 18). Thus, the alterations of Slc7a11 gene are closely related to alterations of Slc7a11 gene, not only in mice but also in human brain cancer patients.

## Discussion

In the current study, we demonstrated a role for Jdp2 in cerebellar lobe formation in mice. Notably, the cerebellum of *Jdp2*-KO mice displayed an altered morphology of lobe formation compared with normal WT cerebellum (Fig. 1). Primary GCPs from *Jdp2*-KO mice expressed lower levels of Atoh1 than WT GCPs [18], indicating that Jdp2 protein might be critical for GCP specificity. Together with the effects observed on primary GCPs, our data suggest that Jdp2 is a cerebellar granule cell marker and a blocker of the differentiation of GCPs to neurons at P5–6.

The aberrant cerebellar lobe formation in *Jdp2*-KO mice could be due to enhanced antioxidant capacity and reduced apoptosis of GCPs during cerebellar development [18], suggesting that ROS may play a critical role in the apoptotic death of GCPs [44]. We found that Slc7a11 expression in Atoh1-positive granule cells was higher in *Jdp2*-KO mouse cerebellum, indicating that Jdp2 is critical for normal development of the cerebellum. However, this relationship of Jdp2 and Slc7a11 expression in GCPs seems to be reversed in Purkinje cells (Fig. 4a). Although we do not know the exact reason for this difference, it might be due to the difference of the higher endogenous ROS levels in Purkinje cells than in GCPs.

Previous studies indicated that in the absence of Jdp2, increase in the expression level of the xCT/Slc7a11 transporter could lead to enhancement of cystine uptake and upregulation of GSH in mice [18]. These results, in turn, indicate that Jdp2 might be a repressor of antioxidant responsive element (ARE) activity in GCPs.

We found that Jdp2 plays a central role in the Nrf2-mediated expression of xCT/Slc7a11 and GSH generation, which inhibits ROS-controlled apoptosis [18, 43]. An excess of antioxidation and lower activity of the ROS content might inhibit programmed cell death in the cerebellum. This indicates that the normal development of the cerebellum is dependent upon high levels of apoptosis of the appropriate cells, especially cerebellar granule cells, which is consistent with the resultant granule cells displaying a lower BrdU incorporation. The lower number of PGCs observed (~ 80%) in *Jdp2*-KO cerebellum compared with WT PGCs (ref. 18), might be due to an inappropriately low level of ROS and ROS-mediated apoptosis activity. Expression levels of marker genes for granule cells were also lower in *Jdp2*-KO GCPs than in WT GCPs. However, the ratios of expression of marker genes in other cell types such as Purkinje cells, astrocytes, and oligodendrocytes were increased 1.6–2.0-fold, suggesting that JDP2 plays a role in the correct differentiation of neural precursors or granule cells into specific cell types, such as Purkinje cells, astrocytes, and oligodendrocytes. Thus, Jdp2 deficiency might stimulate neural differentiation; indeed, our preliminary studies suggest that Jdp2 deficiency contributes to neural differentiation (data not shown).

Further studies are now necessary to elucidate the molecular events associated with the Jdp2-mediated development and differentiation of granule cells.

## Conclusion

A previous study demonstrated that Jdp2 differentially regulated the expression of the *Slc7a11* gene to modulate ROS-mediated neural cell death and cell growth of GCPs. The balance between the static level of Jdp2 and its interaction with Nrf2 in GCPs is essential for the ROS-mediated neural development of these cells in the cerebellum. Our present in vivo data indicate that appropriate Slc7a11 levels in the Atoh1-positive cells and antioxidation activity mediated by Jdp2– Slc7a11–Nrf2 axis are critical factors for the correct development of granule cells in the mouse cerebellum.

## Supplementary Information

**Additional file 1.**

## Data Availability

All data generated or analyzed during this study are included in the published article. All relevant data are available from the authors upon reasonable request. RNA sequencing data were deposited in the NCBI Bioproject Database (http://www.ncbi.nlm.nih.gov/bioproject) with the accession numbers SUB3541857, SUB3541902, SUB3541913, and SUB3541945.

## Abbreviations

ARE: Antioxidant responsive element
BrdU: Bromodeoxyuridine
GCPs: Granule cell progenitor cells
GSH: Glutathione
KO: Knockout
Jdp2: Jun dimerization protein 2
ROS: Reactive oxygen species
Slc7a11: Solute carrier family 7a, member 11
TCGA: The cancer genome atlas
WT: Wild type
