# Supplementary material for: Deletion of Jdp2 enhances Slc7a11 expression in Atoh-1 positive cerebellum granule cell progenitors in vivo
Source: Stem Cell Res Ther. 2021 Jun 29;12:369. doi: 10.1186/s13287-021-02424-4 (PMC8243712; doi:10.1186/s13287-021-02424-4)
Supplement: Supplementary file 1 — Additional file 1. [file 13287_2021_2424_MOESM1_ESM.docx]

**Supplemental Information**

Deletion of *Jdp2* enhances Slc7a11 expression in Atoh-1 positive cerebellum granule cell progenitors *in vivo*

Chia-Chen Ku^1,10^, Kenly Wuputra^1,10^, Kohsuke Kato^2^, Jia-Bin Pan^1,10^, Chia-Pei Li^1,10^, Ming-Ho Tsai^1*^, Michiya Noguchi^3^, Yukio Nakamura^3^, Chung-Jung Liu^4,9,10^, Te-Fu Chan^5^, Ming-Feng Hou^5^, Shigeharu Wakana^6^, Yang-Cheng Wu^7^, Chang-Shen Lin^1^, Deng-Chyang Wu^4,9,10^, and Kazunari K. Yokoyama^1^^,4,10*^

^1^ Graduate Institute of Medicine, Regenerative Medicine and Cell Therapy Research Center, School of Medicine, Kaohsiung Medical University, Kaohsiung 807, Taiwan

^2^ Department of Infection Biology, Graduate School of Comprehensive Human Sciences, the University of Tsukuba, Tsukuba 305-8577, Japan

^3^ Cell Engineering Division, RIKEN BioResource Research Center, Tsukuba 305-0074, Japan

^4.^ Cell Therapy and Research Center, Kaohsiung Medical University Hospital, Kaohsiung 807, Taiwan

^5^ Department of Obstetrics and Gynecology, Kaohsiung Medical University Hospital, Kaohsiung 807, Taiwan

^6^ Japan Mouse Clinic, RIKEN BioResource Research Center, Tsukuba 305-0074, Japan

^7^ Department of Animal Experimentation, Foundation for Biomedical Research and Innovation at Kobe, Hygo 650-0047, Japan

^8^ Chinese Medicine Research and Development Center, China Medical University Hospital , Taichung , Taiwan

^9^ Division of Gastroenterology, Department of Internal Medicine, Kaohsiung University Hospital Kaohsiung 807, Taiwan

^10^ Regenerative Medicine and Cell Therapy Research Center, Kaohsiung Medical University Kaohsiung 807, Taiwan

* ORCID of KK. Yokoyama; 0000-0001-8508-7582 and CS Lin; 0000-0001-7415-2187.

***** Correspondence: [mhtsai1203@hotmail.com](about:blank); [Kazu@kmu.edu.tw](about:blank); Phone; 886-7312-1101, ext. 2729; FAX; 886-7313-3849.

*****Ming-Ho Tsai and Kazunari K. Yokoyama in Graduated Institute of Medicine, Kaohsiung Medical University, 100 Shih-Chuan 1^st^ Road, San-Ming District, Kaohsiung 807, Taiwan; Phone; 886-7312-1101, ext. 2729; FAX; 886-7313-3849; [mhtsai1203@hotmail.com](about:blank); [Kazu@kmu.edu.tw](about:blank)

**Supplementary materials:** The following data are available online.


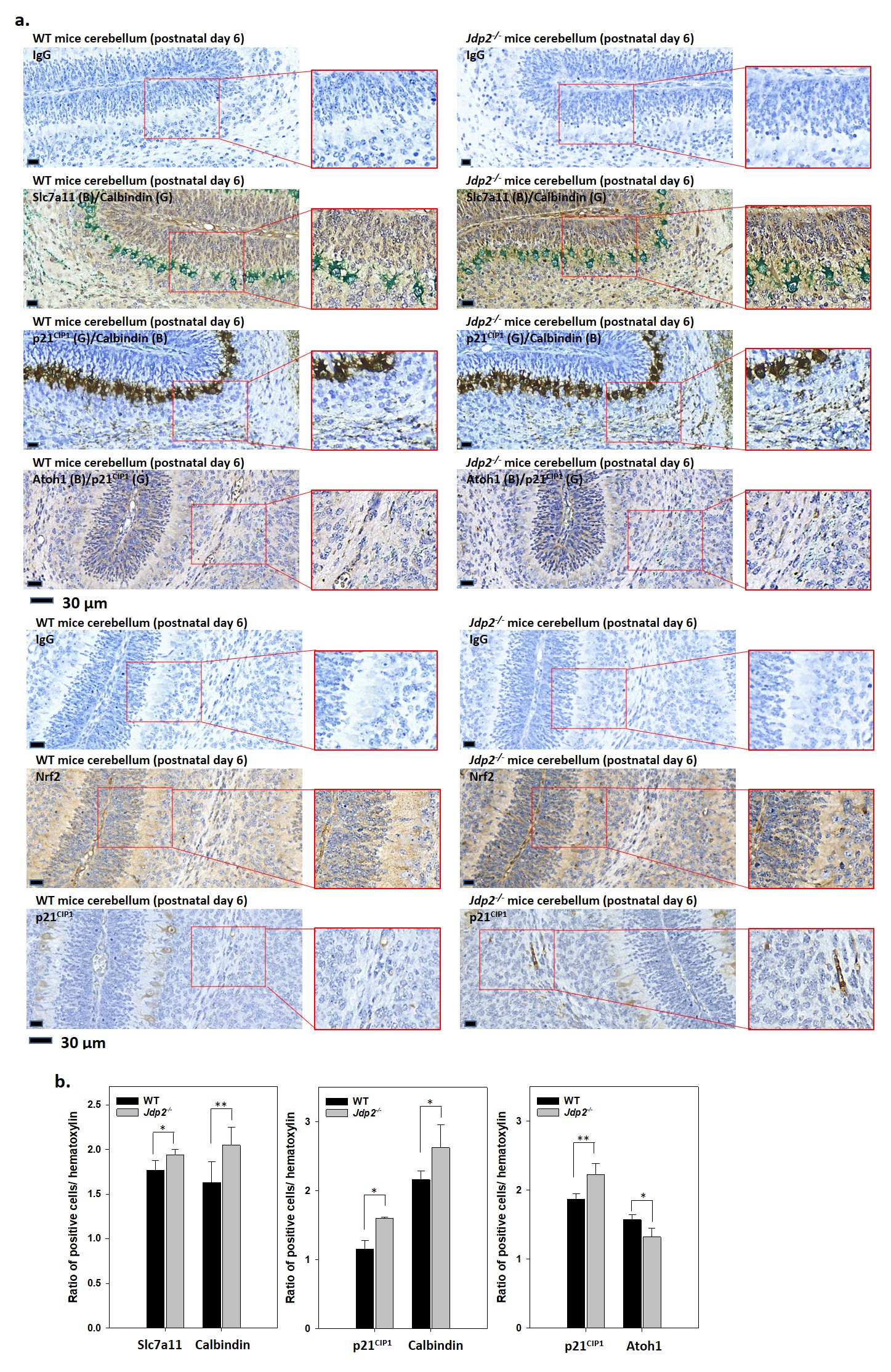


**Supplemental Figure S1. Immunohistochemical analysis of cerebellum in WT and *Jdp2*-KO male mice at P6 was performed using anti-Atoh1, Slc7a11, Nrf2, p21^Cip1^ and Calbindin proteins.**

**a**. Expression of Atoh1, Slc7a11, Nrf2, p21^Cip1^, and Calbindin was detected by immunohistology of the cerebellum in WT and *Jdp2*-KO mice at P6. The section number was 36, the sample number was 16 mice for WT 129-C57/BL6J and 16 mice for *Jdp2*-KO 129-C57/BL6J mice, and lobes IV, and V/VI were shown and whole cerebellar were analyzed. We analyzed each 3 – 4 mice for each sample. **b**. The stained results were quantified as described in Materials and methods. * *p* < 0.05, ** *p* < 0.01.

.


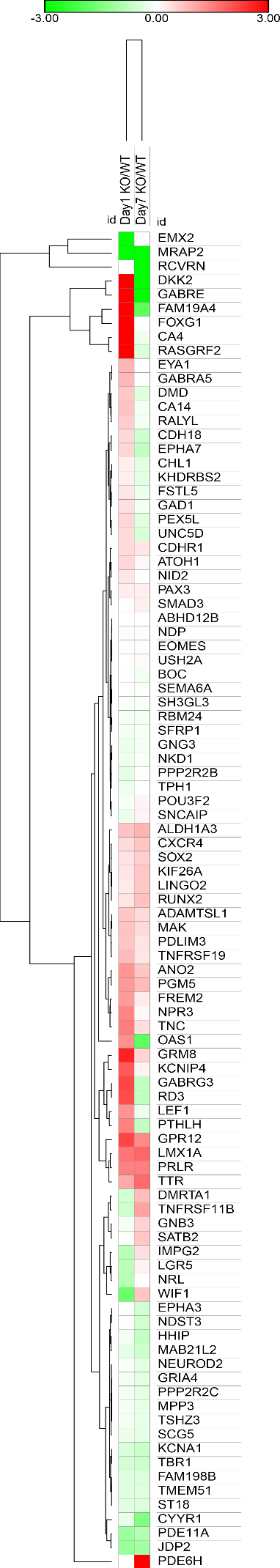


**Supplemental Figure S2. Heatmap of four groups of Medulloblastoma cluster which was combined with the expression profile of Jdp2.**

Heatmap generated from RNA-sequencing data demonstrated the differential expression of genes at higher and the lower levels. The gene expression levels were calculated as reads/kilobase of transcript/million mapped reads (RPKM). Differentially expressed genes were filtered using an RPKM ≥ 0.3, a fold change ≥ 2, and with a *p* value <0.05. RNA sequencing data were deposited in the NCBI Bioproject Database ([http://www.ncbi.nlm.nih.gov/bioproject](about:blank)) with the accession numbers SUB3541857, SUB3541902, SUB3541913, and SUB3541945. Hierarchical clustering of the genes was performed as follows: first, gene-level normalization was performed by transforming the RPKM of each gene of each sample to a Log2 median-centered ratio; subsequently, clustering was obtained by Euclidean distance and complete linkage settings; finally, a heatmap was generated by coloring each gene on the Log2 median-centered ratio. To convert gene symbols to Ensemble gene accessions, the unique gene symbols of each topic were mapped to Ensemble official symbols. A heatmap was shown as coloring each gene on the Log2 median-centered ratio [Log2(KO/WT GCPs)]. i.e. the red color indicates the highest expression, and the green color represents the lowest expression in *Jdp2*-KO GCPs compared with those in WT GCPs. The precise condition was described in the Materials and methods section.


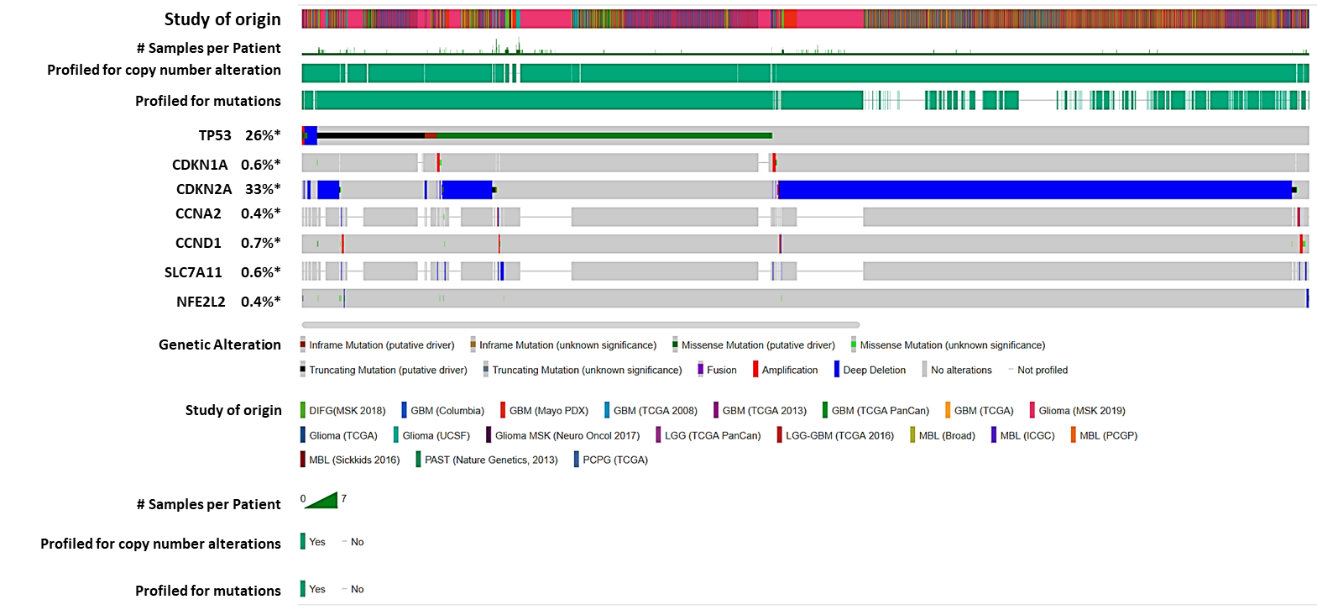


**Supplementary Figure S3**. Representation of mutation maps of Sla7a11-related brain tumors. The eBioPortal ([http://www.cbioportal.org/faq#how-do-i-cite-the-cbioportal](about:blank#how-do-i-cite-the-cbioportal)) data were accessed, and we surveyed Sla7a11 gene mutation maps in brain tumors. 5,952 patients and 6,166 samples from 20 studies were grouped for each item, such as apoptosis and cell cycle progression pathways [33, 34]. We used the cBioPortal database to search for brain-related papers, and we used the filtered 20 papers to query the genes TP53, CDKN1A, CDKN2A, CCNA2, CCND1, SCL7A11, and NFE2L2 in a total of 6,166 samples [32]. Gene alterations of TP53, CDKN1A, CDKN2A, CCND1, SLC7ALL and NFE2L2 were listed. The details were described in the Materials and methods section.


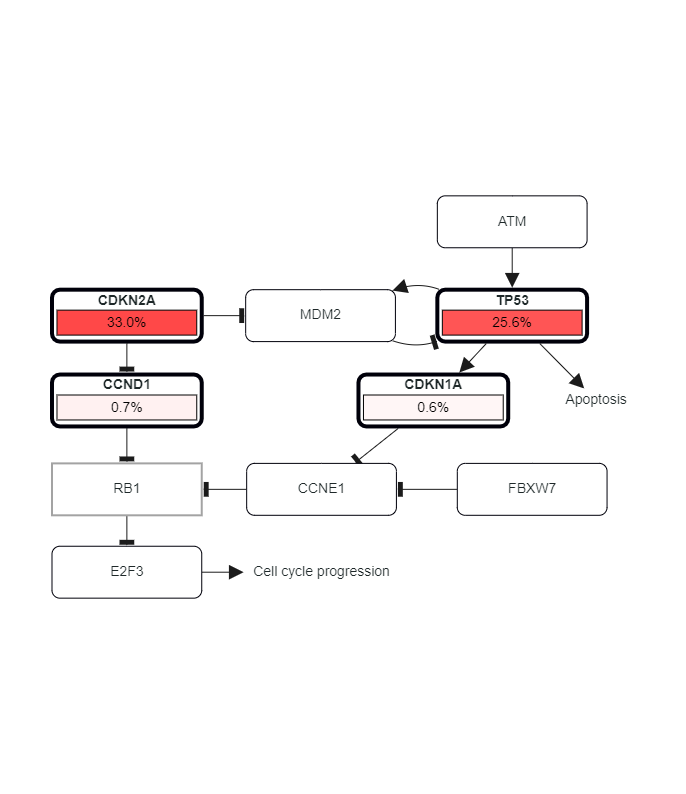


**Supplementary Figure S4**. Presentation of signaling cascade map of Sla7a11-related brain tumors. Apoptosis and cell cycle progression pathways are presented by the data in SFig. 3. 5,952 patients and 6,166 samples in 20 studies were summarized in cBioPortal for Cancer Genomics [32]. TP53 and CDKN2A were tremendously mutated in these brain cancers. The details were described in the Materials and methods section.


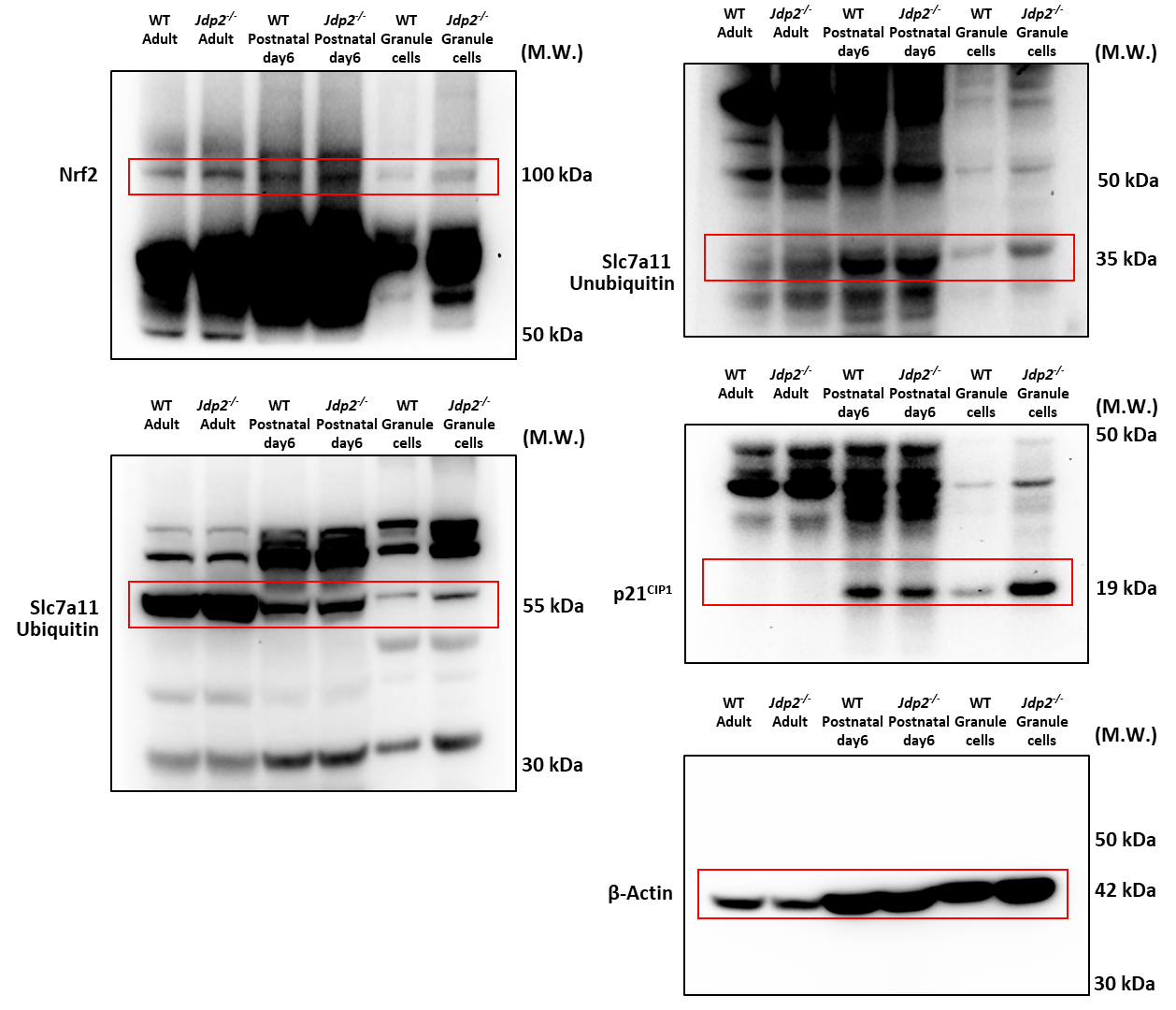


**Supplementary Figure S5.** The full-length western blotting data of the cerebella from WT and Jdp2-KO mice (9 weeks, and P6), and GCPs derived from WT and Jdp2-KO mice (P6). The red closed lines indicate the spliced region of the whole data. The western blotting was performed as described in Materials and methods. M.W.; molecular weight. Slc7a11 were presented as the ubiquitinated protein (55 kDa) and unubiquitinated protein (35 kDa) as shown in ref. 18.

**Supplementary Table S1**. Presentation of co-occurrence tendency ratio of Sla7a11-related brain tumors. 5,952 patients and 6,166 samples in 20 studies were summarized in cBioPortal for Cancer Genomics [32]. SC7A11expression was strongly correlated with CCNA2, TP53, and CDKN1A. The details were described in the Materials and methods section.

**Supplementary Table S2.** Summary of the data of cBioPortal for Cancer Genomics. Percentages of alteration in profiled, total number of mutations, number of samples with one or more mutations, profiled samples, and frequency of the alteration were listed. 5,952 patients and 6,166 samples in 20 studies were summarized in cBioPortal for Cancer Genomics [32]. The details were described in the Materials and methods section.
